# Technik der Diagnose, Operation und Harnleiterbehandlung bei Nierentuberkulose

**Published:** 1913-09

**Authors:** 


**Technik der Diagnose, Operation und Harnleiterbehandlung bei Nierentuberkulose.** Von Hofrat Dr. FELIX SCHLAGINTWEIT. S. v, 143. München: J. F. Lehmann. 1912.

Since 1906 Dr. Schlagintweit has made a special study of tuberculosis of the kidney, and given minute attention to technique in diagnosis, operation, and treatment of the ureter during the operation and subsequently. The results of this study are given to the reader in the book before us.

First the origin, the seat of the primary focus and the mode of spread of urogenital tuberculosis are considered, then the symptoms, then the technique employed in diagnosis, and lastly the operation of nephrectomy in all its details. Case histories are freely cited to illustrate special points. The sections in which the above matters are dealt with are each and all interesting and illuminating.

The author is a strong supporter of early nephrectomy, and even of late nephrectomy if the opportunity for early operation has been allowed to pass away.

A large portion of the book is devoted to a consideration of the treatment of the ureter—a vexed question. Hard and fast rules for treatment of the ureter are not laid down, but various methods which the author has tried are described, and those methods which deserve further trial in extended series of cases are indicated.

It would be easy to quote from the pages of this book many valuable observations and conclusions, but we must instead strongly advise surgeons to read what is there written for themselves. The systematised method of investigation advocated is worthy of the closest imitation.

The possibility of early diagnosis and the danger of delay in the treatment of urogenital tuberculosis should be better understood by practitioners. As the author points out, few things can be simpler for a practitioner to do than to send a speciment of urine to a pathological institution to be examined for tubercle by inoculation if ordinary examination yields a negative result. This should be done without delay in all cases in which renal or vesical troubles are not obviously due to other causes then tubercle. Chiefly in that way will it be possible to diminish the number of those terrible cases of vesical tuberculosis with which we are all only too familiar.

